# Unique perception of clinical trials by Korean cancer patients

**DOI:** 10.1186/1471-2407-12-594

**Published:** 2012-12-12

**Authors:** Su Jin Lee, Lee Chun Park, Jeeyun Lee, Seonwoo Kim, Moon Ki Choi, Jung Yong Hong, Sylvia Park, Chi Hoon Maeng, Wonjin Chang, Young Saing Kim, Se Hoon Park, Joon Oh Park, Ho Yeong Lim, Won Ki Kang, Young Suk Park

**Affiliations:** 1 Division of Hematology/Oncology, Department of Medicine, Samsung Medical Center, Sungkyunkwan University School of Medicine, 50 Irwon-dong Gangnam-gu, Seoul, 135-710, Korea; 2 Department of Internal Medicine, Kosin University Gospel Hospital, Busan, Korea; 3 Biostatistics team, Samsung Biomedical Research Institute, Samsung Medical Center, Seoul, Korea

**Keywords:** Cancer clinical trial, Survey, Awareness and willingness to participate

## Abstract

**Background:**

In the past few years, the number of clinical trials has increased rapidly in East Asia, especially for gastric and hepatobiliary cancer that are prevalent in Asian populations. However, the actual degree of understanding or perceptions of clinical trials by cancer patients in East Asian countries have seldom been studied.

**Methods:**

Between July 1^st^ and November 30^th^ of 2011, we conducted a prospective study to survey cancer patients regarding their awareness of, and willingness to participate in, a clinical trial. Patients with gastrointestinal/hepatobiliary cancer who visited the Hematology-Oncology outpatient clinic at Samsung Medical Center (SMC) were enrolled. A total of 21 questions were asked including four questions which used the Visual analogue scale (VAS) score.

**Results:**

In this survey study, 1,000 patients were asked to participate and 675 patients consented to participate (67.5%). The awareness of clinical trials was substantially higher in patients who had a higher level of education (*p*<0.001), were married (*p*=0.004), and had a higher economic status (*p*=0.001). However, the willingness to participate in a clinical trial was not affected by the level of education or economic status of patients. The most influential factors for patient willingness to participate were a physician recommendation (n=181, 26.8%), limited treatment options (n=178, 26.4%), and expectations of effectiveness of new anti-cancer drugs (n=142, 21.0%). Patients with previous experience in clinical trials had a greater willingness to participate in clinical trials compared to patients without previous experience (*p*<0.001).

**Conclusions:**

This large patient cohort survey study showed that Korean cancer patients are more aware of clinical trials, but awareness did not translate into willingness to participate.

## Background

Oncology is one of the most rapidly developing fields in medicine and a greater understanding of tumours at the molecular level has resulted in the development of many novel therapeutic agents. Cancer clinical trials are needed to evaluate the efficacy and toxicity of these newly-developed agents and have become an essential component of oncology [1,2]. Besides the design of the trial and the efficacy of the drug, the key success factor to conducting an effective clinical trial is an understanding and awareness of clinical trials by cancer patients. Patient attitude towards a clinical trial could greatly influence patient recruitment and retention. Although general attitudes towards clinical trials are positive in Western Countries, the number of patients actually enrolled in clinical trials tends to be low [3-7]. The known barriers to successful clinical trials include changes in hospital administrative systems, fewer eligible patients than anticipated (inaccurate feasibility), problems inherent in certain types of trials or methodology (e.g. placebo control), no treatment arms (supportive care only), and difficulties in describing randomization [8-10]. One of the major patient-related factors affecting the success of clinical trials is a lack of understanding of or low awareness by the patients themselves, which may impede patient accrual. Hence, it is necessary to understand the perception of clinical trials by cancer patients.

Previous studies have used questionnaires to examine patient expectation and understanding of clinical trials, to elucidate existing barriers and to improve the processes of clinical trials [11-14]. The National Cancer Institute (NCI) estimated that less than 5% of newly diagnosed adult cancer patients are enrolled in clinical trials in the United States [1]. In Asia, even though the number of clinical trials has increased rapidly in the past decade, especially for cancer types prevalent in the Asian population, the awareness, understanding and/or willingness to participate has scarcely been reported. In fact, Korea leads Asia in patient recruitment for cancer trials and is 10^th^ in the world [15]. Owing to a relatively short history of cancer clinical trials and cultural differences in the doctor-patient relationship, Western data on patient attitude may not be directly applicable to such Eastern populations. In addition, cancer type could also affect the perception of and participation in a clinical trial, but few studies have addressed these questions [16].

The aim of this study was therefore to survey and analyze the degree of understanding and perceptions of clinical trials in Korea.

## Methods

### Data collection

From July 1^st^ to November 30^th^ of 2011, we surveyed cancer patients who were diagnosed with gastrointestinal cancer (including gastric, colorectal, esophageal, small intestinal, hepatobiliary, and pancreatic cancer) at Samsung Medical Center in Korea and who visited the outpatient clinic at the Division of Hematology-Oncology. The eligibility criteria were: 1) aged >19 years; 2) histologically confirmed gastrointestinal or hepatobiliary-pancreatic cancer; and 3) signed informed consent.

One well-trained research nurse explained the purpose of the survey and received written informed consent from all participants before they were enrolled in the study. All participants completed the questionnaire by themselves. With the agreement of the participants, clinical information of each patient was collected from medical records. This study was approved by the Institutional Review Board of the Association for the Accreditation of Human Research Protection Program, approved by Samsung Medical Center. The study was carried out in accordance with the tenets of the Declaration of Helsinki.

### Questionnaire

The questionnaire consisted of 21 questions based on an Index of Clinical Trial Understanding [17], and included questions that used a Visual analogue scale (VAS) score, as well as single- and multiple-choice questions. The survey was developed in collaboration with the Biostatistics Core at Samsung Medical Center. A VAS between 0 and 10 was used in the survey to evaluate the level of awareness of clinical trials and participation in them. A VAS score of zero indicated ‘never’ and 10 indicated ‘absolute’. Eight of the questions concerned demographics such as age, sex, travel distance from the hospital, level of education, residence, marital status, economic status, and religion. Eight more questions asked about cancer clinical trial understanding (awareness) based on an Index of Clinical Trial Understanding [17], and the remaining five questions were about willingness to participate in clinical trials (Additional file 1).

### Statistical analyses

The Kruskal-Wallis rank sum test and the Mann–Whitney test were used in the analysis of awareness and willingness according to patient characteristics. Fisher's Exact Test for Count Data was used, depending on the presence or absence of clinical trial experience. The VAS score with multiple factors (answers for single-choice or multiple-choice questions) was analyzed by Kruskal-Wallis rank sum test or Kruskal-Wallis chi-squared test. VAS scores of both groups were analyzed by Spearman correlation analysis. A biostatistician was consulted for all statistical designs and analyses and all tests were considered statistically significant at a *p*< 0.05.

## Results

### Patient cohort

From July 1^st^ to November 30^th^ of 2011, a total of 1,000 patients were asked to participate in the study, and 675 patients (67.5%) agreed to complete the questionnaire. Patients who were diagnosed with gastrointestinal or hepatobiliary-pancreatic cancer (gastric, colorectal, esophageal, small intestinal, hepatobiliary, pancreatic cancer) at Samsung Medical Center in Korea were enrolled in the study. Table 1 shows the participant characteristics at baseline. The age of the participants ranged widely from 31 to 86 years, with a median age of 60 years. More patients were male than female (n=438, 64.9% vs. n=237, 35.1%). High school was the most frequent level of education (47.0%) and most participants were married (90.8%). Colorectal cancer was the most common cancer type (n=341) followed by gastric cancer (n=191), hepatobiliary cancer (n=57), pancreatic cancer (n=51), esophageal cancer (n=25), and small bowel cancer (n=10). In terms of the disease status, 254 patients (37.7%) were in first-line palliative chemotherapy, 250 (37.0%) were in neoadjuvant or adjuvant chemotherapy and the remaining patients (N=168, 24.9%) were in more than second-line chemotherapy. A total of 127 participants (18.8%) had a prior history of clinical trial enrollment; the remainder had no prior exposure to clinical trials.

**Table 1:** Participant characteristics at baseline

**Variables**		**Frequency (N=675)**	**%**
Age	Median(years)	60 ± 10	
Sex	Male	438	64.9
	Female	237	35.1
Travel time (to Hospital from home)	<2 hours	363	53.8
	≥2 hours	312	46.2
Place of residence	Seoul/Gyeonggido	325	48.1
	Chungcheongdo	125	18.5
	Gyeongsangdo	146	21.6
	Jeollado	48	7.1
	Gangwondo	21	3.1
	Jejudo	10	1.5
Education level	≤Middle school	154	22.8
	High school graduate	317	47.0
	≥College graduate	204	30.2
Marital status	Married	613	90.8
	Single	17	2.5
	Widowed/Divorced	45	6.7
Economic status (Income/month)	≥5.000.000 won*	89	13.2
	2.000.000~5.000.000won	308	45.6
	≤2.000.000 won	278	41.2
Religion	Christian	175	25.9
	Catholic	69	10.2
	Buddhist	196	29.0
	No religion	231	34.2
	Other	4	0.6
Cancer type	Gastric	191	28.3
	Colorectal	341	50.5
	Esophagus	25	3.7
	Small intestinal	10	1.5
	Hepatobiliary	57	8.4
	Pancreatic	51	7.6
Disease status	Neoadjuvant or adjuvant	250	37.0
	Palliative ≤ first-line	257	38.1
	Palliative ≥ second-line	168	24.9
Prior history of clinical trial enrollment	Yes	127	18.8
	No	548	81.2

*1000 won is equivalent to one dollar.

### Awareness of clinical trials

The mean score responses to the question ‘On a scale of 0 to 10, how would you rate your understanding of cancer clinical trials?’ was 4.25 ± 2.5 and nearly half of the participants (47.9%) selected a value of 5. Table 2 shows the relationship between awareness of clinical trials and patient baseline characteristics. A higher educational degree (*p*<0.001), being married (*p*=0.004), and higher economic status (*p*=0.001) were associated with a higher rate of awareness of clinical trials, whereas sex, travel distance from hospital, residing province in Korea, and type of religion were not significantly associated with clinical trial awareness.

**Table 2:** Patient awareness of clinical trials and willingness to participate

**Variable**		**Awareness (P-value)**		**Participation (P-value)**	
Sex	Male	4.4	0.082	5.2	0.011
	Female	4.0		4.6	
Distance from hospital	<2 hour	4.3	0.919	5.0	0.976
	≥2 hour	4.2		5.0	
Place of residence	City of Seoul/Suburb	4.2	0.895	4.9	0.865
	Chungcheongdo	4.4		5.3	
	Gyeongsangdo	4.1		5.0	
	Jeollado	4.3		5.1	
	Gangwondo	5.0		5.0	
	Jejudo	4.1		4.2	
Highest level of education	≤ Middle school	3.2	<0.001	4.6	0.286
	High school	4.3		5.0	
	≥ University	5.0		5.2	
Marital status	Married	4.4	0.004	5.0	0.685
	Single	3.2		5.2	
	Widowed/Divorced	3.2		4.5	
Economic status (Income/month)	≥5.000.000 won	5.0	0.001	5.4	0.310
	2.000.000~5.000.000won	4.4		5.0	
	≤2.000.000 won	3.8		4.8	
Religion	Christian	4.2	0.552	5.4	0.078
	Catholic	4.6		4.4	
	Buddhist	4.1		5.2	
	No religion	4.3		4.7	
	Other	6.5		5.0	

### Cancer clinical trial understanding

Cancer clinical trial understanding was measured using a revised Index of Clinical Trial Understanding (ICTU) [17]. When asked about their knowledge of a control group (Q#10, Table 3), more than 50% of patients (361 of 695) were aware that a randomized clinical trial was the most appropriate method to prove the efficacy of a new cancer drug. More than 80% of patients (545 of 675) agreed (VAS score > 5) that a cancer clinical trial tested a new drug by randomizing patients to novel treatment vs. placebo (Q#14, mean VAS = 6.1 ± 3.1). Nevertheless, when questioned about their knowledge of random assignment, 214 of 695 patients (31.7%) answered that a treatment group can be selected based on the patient’s preference in a randomized trial. Each respondent was subsequently asked about the meaning of random assignment. Approximately 80% of the patients answered that they will be randomly assigned according to the severity of their disease or by a physician’s decision. When asked about their knowledge of standard therapy, 45% of patients (317 of 675) understood the meaning of standard treatment to be the best available therapy for that particular cancer and 15% (106 of 675) regarded standard treatment as an average cancer therapy. Knowledge of placebo was tested using a VAS score (0, completely disagree to 10, completely agree). Item Q#14 on the questionnaire asked how much participants agreed with the following statement: “When new cancer drugs are tested, half of the subjects always receive a placebo instead of the new medicine.” Item Q#15 provided a measure of the general understanding of the purpose of a clinical trial and asked how much participants agreed with the following statement: “Clinical trials are essential to advancing medical science and improving the standard of care.” In general, cancer patients were well aware of the necessity and purpose of cancer clinical trials with a mean VAS score of 8.1 ± 2.5. The most frequent source for obtaining information about clinical trials was a physician (n=391, 52%) followed by mass media (n=273, 36.6%), other patients (n=52, 6.9%), and the internet (n=25, 3.3%).

**Table 3:** Cancer clinical trial understanding

**Question items**	**answers**	**N**	**%**
Q#10 Which method do you think would be more effective in determining the effectiveness of the new drug?	1. Give the new drug to 1000 newly diagnosed cancer patients.	100	14.8
	2. Give the new drug to 500 newly diagnosed cancer patients and give the currently used drug to 500 other newly diagnosed patients and see which group improves the most.	361	53.5
	3. Not sure	214	31.7
Q#11 Randomization	1. Patients should be allowed to select the test group that they want.	214	31.7
	2. Patients need to be assigned to a test group randomly to make the test accurate.	167	24.7
	3. It doesn’t make any difference; either approach will work.	106	15.7
	4. Not sure	188	27.9
Q#12 If a doctor were to tell you that patients in a clinical trial were to be assigned to the new therapy and the standard therapy randomly, do you understand randomly to mean that patients would be assigned by:*	1. The severity of their disease.	235	33.0
	2. An independent factor such as the two last digits of a patient’s telephone number.∗	38	5.3
	3. The kind of health insurance that they have.	14	2.0
	4. The doctor depending on which group needs more participants.	311	43.7
	5. I am not sure what “randomly” means.	114	16.0
Q#13 If a doctor were to tell you that patients in a clinical trial were to be assigned to the new therapy and the standard therapy randomly, do you understand standard therapy to mean:*	1. An average cancer therapy, not the best and not the worst.	106	15.0
	2. The best available therapy for that particular cancer.	317	44.9
	3. A placebo or sugar pill with no medical value.	5	0.7
	4. An experimental therapy involving a new drug for cancer.	79	11.2
	5. I am not sure what “standard therapy” means.	199	28.2

*Multiple selections were allowed.

### Participation in clinical trials

The VAS score evaluation of the willingness to participate in clinical trials produced a mean score of 5.0 ± 3.1. The total score had an even distribution from 0 to 10 and 256 patients (37.9%) selected 5 as the VAS score. Table 2 shows the relationship between willingness to participate in clinical trials and patient characteristics at baseline. Men were more likely to participate in clinical trials than women (*p*=0.001). Importantly, there was a discrepancy between awareness and participation in clinical trials. Patients with a higher level of education that were married and had a higher income had a better understanding of clinical trials. However, the understanding of clinical trials did not directly translate into willingness to participate. Willingness was not affected by level of education (*p*=0.286) or economic status (*p*=0.310). Of note, patients who obtained their information from physicians were more likely to participate in clinical trials (*p*<0.001), whereas patients who acquired information from mass media were less likely to participate (Table 4, *p*=0.027). The most influential person involved in the decision to participate in clinical trials was “patient him- or her-self” (n=473, 62.4%), followed by ”family” (n=183, 24.1%). A common reason for cancer trial participation was physician recommendation (n=181, 26.8%) and no other treatment options (n=178, 26.4%). Nevertheless, the patients who selected these two answers showed a lower level of willingness to participate. Conversely, patients who provided exposure to a new drug as the most influential factor for participation demonstrated the highest likelihood of clinical trial participation (6.141, *p*<0.001). The most important reason for refusal to participate was unconfirmed treatment (n=320, 47.4%) followed by negative perception of clinical trials per se (*p*=0.035). Patients with a negative perception of clinical trials showed the lowest willingness to participate.

**Table 4:** Willingness to participate in a clinical trial

**Question**	**Answers**	**N(%)**	**Participation in a clinical trial**	**p-value**
Source of information about clinical trials*	Physician	391(52.0)	5.4	<0.001
	Mass media	273(36.6)	4.7	0.027
	Other patients	52(6.9)	4.8	0.696
	Internet	25(3.3)	5.3	0.642
	Other	11(1.5)	3.7	0.195
Prior participation to clinical trials	Yes	127(18.8)	6.8	<0.001
	No	548(81.2)	4.6	
The decision-making person for clinical trial enrollment*	Self	473(62.4)	5.1	0.298
	Husband, wife	61(8.0)	5.5	0.187
	Children	28(3.7)	4.1	0.150
	Parents	1(0.1)	5.0	0.968
	Family council	183(24.1)	4.8	0.345
	Other	12(1.6)	6.0	0.269
Most influential factor for clinical trial participation	Exposure to a new drug	142(21.0)	6.1	<0.001
	Financial benefit	50(7.4)	5.3	
	Contribution to medicine	119(17.6)	5.7	
	Physician’s recommendation	181(26.8)	4.4	
	No other treatment option	178(26.4)	4.1	
	Other reasons	5(0.7)	7.6	
Most influential factor for refusal to participate	Negative perception of clinical trials	193(28.6)	4.5	0.035
	Unconfirmed treatment	320(47.4)	5.2	
	Frequent hospital visits	53(7.9)	5.8	
	Prefers standard treatment	101(15.1)	4.9	
	Other reasons	8(1.2)	5.8	

### Comparison of patients with and without experience of clinical trials

Among the 675 patients, 127 (18.8%) had prior experience of cancer clinical trial participation (Table 5). Patients with previous experience in clinical trials were more willing to participate in clinical trials compared to patients without previous experience in clinical trials (*p*<0.001).

**Table 5:** Prior exposure to clinical trials and willingness to participate

**Question**	**Answers**	**Patients in previous clinical trials (N=127) (%)**	**Patients never in clinical trial (N=548) (%)**	**p-value**
Most influential factor for clinical trial participation	Using a new drug	39(30.7)	103(18.8)	0.001
	Economic benefit	8(6.3)	42(7.7)	
	Contribute to medical development	21(16.5)	98(17.9)	
	Physician’s recommendation	37(29.1)	144(26.3)	
	No other treatment option	19(15.0)	159(29.0)	
	Other	3(2.4)	2(0.4)	
Most influential factor for refusal to participate	Negative perception of clinical trials	28(22.0)	165(30.1)	0.019
	Unconfirmed treatment	65(51.2)	255(46.5)	
	Frequent hospital visits	8(6.3)	45(8.2)	
	Prefers standard treatment	21(16.5)	80(14.6)	
	Other reasons	5(4.9)	3(0.6)	

For the question “What is the most important reason for participating if you decide to join a clinical trial?”, “using a new drug” (n=39, 30.7%) ranked first for patients with experience of clinical trials, whereas “for nothing other than treatment” (n=159, 29.0%) was the leading cause in patients without experience of clinical trials. For the question “What is most important reason for not participating if you decide not to join a clinical trial?”, “the anxiety of unverified treatment” (51.2%, 46.5%, respectively) was the most common answer in both groups. The second most common answer was “objection to clinical trials themselves” (22.0% vs. 30.1%) and the difference between the two groups was statistically significant (*p*=0.019).

## Discussion

Korea has been facing a rapid increase in cancer incidence [18], and the volume of cancer clinical trials has increased accordingly. Progress in cancer treatment can only come from investigations of novel therapies and treatment strategies performed in the setting of well conducted clinical trials [1]. Because participation in clinical trials has frequently been associated with a higher survival rate [19], clinical trials have become an important facet of oncology, and the need for clinical studies continues to grow. Our study has shown that willingness to participate in clinical trials is intermediate in Asian patients with GI and hepatobiliary-pancreatic cancer. The mean VAS score of 5.0, the median score of 5, and evenly distributed total score from 0 to 10 showed some differences from results of prior studies performed in Western countries, where the majority of patients with cancer were willing to consider participating in clinical trials [14,17,20,21].

Overall, Korean cancer patients understood the need for two treatment arms to prove drug efficacy (N=361, 53.5%), which is considerably higher than in the Western studies (33% of the public and 37% of cancer survivors) [17]. Nevertheless, our study indicated that more than 70% of patients did not possess accurate knowledge of randomized trials: 31.7% (n=214) of patients thought they could choose the study treatment arm in a randomized trial and 43.7% (n=311) answered that a doctor’s decision is the most influential factor for randomization. Only 5.3% of patients understood the exact meaning of randomization, which is substantially lower than the percentage reported in Western studies (63% of the public and 67% of cancer survivors). Furthermore, less than half of our cancer patients were aware that standard treatment referred to the best current practice for a specific disease while 66% of the Western cancer patients were aware of the meaning of standard treatment in cancer care [17]. Taken together, although a higher rate of cancer patients were aware of randomized trials than in the West, a much smaller percentage of cancer patients had precise knowledge of the randomization process or of assignment of a treatment arm in randomized trials. Based on our results, more detailed explanations on the randomization procedure should be provided to patients at our hospital, since there was a high discordance rate between the awareness and the understanding of a randomized clinical trial.

Interestingly, a higher level of awareness did not always result in increased willingness to participate in clinical trials, in contrast to the findings of previous Western studies [4,7,21-24]. This might represent one of the peculiar features of Asian cancer patients that should be confirmed in another cancer patient cohort.

As shown in Tables 4 and 5, the most influential factor for willingness to participate in clinical trials was ‘physician’s recommendation’ (26.8%) and this was similar to Western findings which showed the strongest predictor of expected enrollment of clinical trials was encouragement by the physician [17]. In contrast to a common belief, however, the main decision-making person for clinical trial participation in Korea was the patient (473 of 675, 62.4%).

In this study cohort, a total of 127 participants (18.8%) had a prior history of clinical trial enrollment. This population represented a unique patient population with different behavior from the remaining patients. For instance, there was high concordance between clinical trial awareness and willingness to participate (*p*<0.001). In addition, the most common motivation for clinical trial participation was exposure to a new drug (30.7% vs. 18.8%; prior experience vs. no experience group, Table 5). In addition, the negative perception of clinical trials was significantly lower in patients (n=28 of 127, 22%) with prior experience when compared with no experience (n=165 of 548, 30.1%).

We note that our study has some limitations. First, an inherent selection bias may exist owing to the fact that the study was conducted in only one tertiary academic hospital. Second, another selection bias was introduced because our data did not include all 1000 patients who were asked to join this survey and only 67.5% of patients completed the questionnaire. In addition, the percentage of clinical trial participants may have influenced and altered the results of this survey. The rate of clinical trial participants for GI and hepatobiliary-pancreatic cancer at Samsung Medical Center ranges from 10 – 20% at all times which is in accordance with the proportion of clinical trial participants in this study (18.8%). Hence, the proportion of participants was not biased or misrepresented in this study.

## Conclusions

In conclusion, our study involved a large patient cohort and we used VAS scores to inquire about the awareness of, and willingness to participate in, clinical trials in Asian cancer patients. Based on this study, we plan to do a follow-up study in five or ten years to analyze the evolving trend in perception of clinical trials in Korean cancer patients. Through this survey study, we established and defined unique features of our patients. Understanding a cancer patient’s perception, willingness to participate, and influential factors underlying decisions could facilitate patient recruitment for clinical trials in Asia.

## Competing interests

The authors declare that they have no competing interests.

## Authors’ contributions

JL, SHP, JOP, HYL, WKK and Y SP contributed substantially to the conception and design of this study. MKC, JYH, SP, CHM, WC and YSK contributed to data acquisition. SK analyzed data. SJL and LCP interpreted data and prepared a draft manuscript. JL and YSP produced a final draft and provided approval for submission of this manuscript. All authors read and approved the final manuscript.

## Pre-publication history

The pre-publication history for this paper can be accessed here:

http://www.biomedcentral.com/1471-2407/12/594/prepub

## Supplementary Material

Additional file 1 Questionnaire. Click here for file
