# Supplementary material for: Unique perception of clinical trials by Korean cancer patients
Source: BMC Cancer. 2012 Dec 12;12:594. doi: 10.1186/1471-2407-12-594 (PMC3572433; doi:10.1186/1471-2407-12-594)
Supplement: Additional file 1 — Questionnaire. [file 1471-2407-12-594-S1.docx]

Supplementary (Questionnaire)

Q1. Age : ________________

Q2. Sex : Female ( ) Male ( )

Q3. What is the travel time from your home to the hospital?

① Less than two hours

② Two or more hours

Q4. Where is your place of residence?

① Seoul/Gyeonggido

② Chungcheongdo

③ Gyeongsangdo

④ Jeollado

⑤ Gangwondo

⑥ Jejudo

Q5. What is your highest level of education?

① Middle school or below

② High school

③ College or above

Q6. What is your marital status?

① Married

② Single

③ Widowed/Divorced

Q7. What is your monthly household income?

① 5,000,000 won or more

② Between 2,00,0000 and 5,000,000 won

③ Less than 2,00,0000 won

Q8. What is your religious affiliation?

① Christian

② Catholic

③ Buddhist

④ No religion

⑤ Others( )

II. Level of Awareness

Please check the box that applies to you or elaborate in case of “Others”.

Q9. If you would be informed of the cancer clinical trial via internet or other media (e.g. newspaper or television), how well do you believe you understand the clinical trial? (Circle the level of your understanding.)


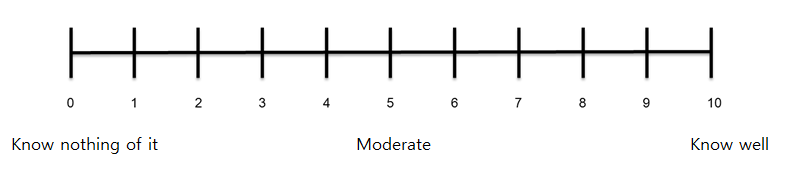


Q10. Which method do you think would be more effective in determining the effectiveness of the new drug?

① Give the new drug to 1000 newly diagnosed cancer patients.

② Give the new drug to 500 newly diagnosed cancer patients and give the currently used drug to 500 other newly diagnosed patients and see which group improves the most.

③ Not sure.

Q11. If there would be two test groups, should the patients be allowed to select the test group they want? Or should the patients be assigned to a test group randomly?

① Patients should be allowed to select the test group that they want. (New therapy and standard therapy)

② Patients need to be assigned to a test group randomly to make the test accurate

③ It doesn’t make any difference; either approach will work.

④ Not sure.

Q12. If a doctor were to tell you that patients in a clinical trial are to be assigned to either the new therapy or the standard therapy randomly, your understanding of “randomly assigned therapy” would be determined by which of the following? (Please check all that applies)

① The severity of their disease.

② An independent factor such as the two last digits of a patient’s telephone number.

③ The kind of health insurance that they have.

④ The doctor depending on which group needs more participants.

⑤ I am not sure what “randomly” means.

Q13. If a doctor were to tell you that patients in a clinical trial are to be assigned to either the new therapy or the standard therapy randomly, which of the following would be your understanding of standard therapy? (Please check all that applies.)

1. An average cancer therapy, not the best and not the worst.

② The best available therapy for that particular cancer.

③ A placebo or sugar pill with no medical value.

④ An experimental therapy involving a new drug for cancer.

⑤ I am not sure what “standard therapy” means.

Q14. How much do you agree that a half of the cancer clinical trial participants are randomly assigned to a placebo group?


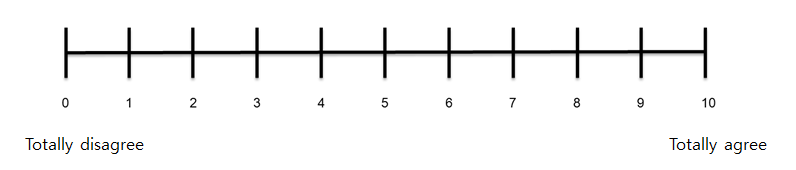


Q15. Cancer clinical trials are critical to advancing medicine and improving therapy effectiveness. How much do you agree to this?


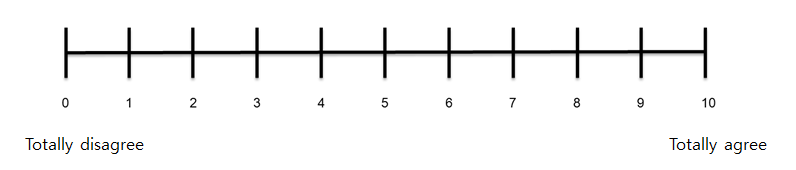


Q16. What was your source of information about clinical trials? Please check all that applies.

① Physicians or medical professionals

② Mass media

③ Other patients

④ Internet

⑤ Others( )

III. Willingness to participate

Please check all that applies or elaborate in case of “Others”.

Q17. Do you have prior experience of clinical trials?

① Yes.

② No.

Q18. How much are you willing to participate in a cancer clinical trial if you were proposed to one now?


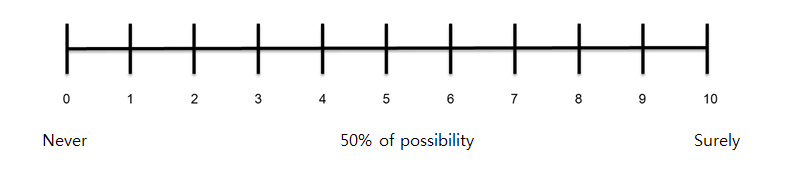


Q19. Who is the decision making person for clinical trial enrollment? Please check all that applies.

① Self

② Spouse

③ Children

④ Parents

⑤ Family council

⑥ Others( )

Q20. What is **the most influential factor** for clinical trial participation? (Please check one only.)

① Exposure to a new drug

② Financial benefit

1. Contribution to medicine

④ Physician’s recommendation

⑤ No other treatment option

⑥ Others ( )

Q21. What is **the most influential factor** for refusal to participate? (Please check one only.)

① Negative perception of clinical trials

② Fear of unconfirmed treatment

③ Frequent hospital visits

④ Prefer standard treatment

⑤ Others ( )
